# Effects of surgical factors on the outcomes of zygoma reduction malarplasty: a quantitative computed tomography study

**DOI:** 10.1186/s40902-023-00371-z

**Published:** 2023-01-09

**Authors:** Jong Chul Park

**Affiliations:** Wonjin Dental Clinic, Pagoda Tower 1306 6 Seocho-dong Seocho-gu, Seoul, South Korea

**Keywords:** Zygoma reduction malarplasty, Computed tomography, Multiple regression analysis, Pre-bending plate, Bone segmentation

## Abstract

**Background:**

Malarplasty is widely performed for zygoma reduction. The effects of body segmentation, plate bending, and postoperative arch location on zygomatic movement have not been analyzed using computed tomography (CT).

**Results:**

We quantitatively analyzed the effects of surgical factors on zygomatic movements via superimposition of preoperative and postoperative CT images using three-dimensional software. Our results showed that segmentation had the most significant effect on the horizontal reduction of malar eminence (*β* = 0.593, *r* = 0.696, adjusted *r*^2^ = 0.479, *F* = 79.595; *p* < 0.001). In addition, upward and posterior arch movements had significant effects on the anterior and posterior movements of the eminence (*β* = − 0.379 for vertical arch movement, *β* = 0.324 for arch setback, *r* = 0.603, adjusted *r*^2^ = 0.352, *F* = 31.943; *p* < 0.001). The major factors that influenced inward arch movement at the coronoid process included segmentation and inward movement at the arch osteotomy site. To prevent interference of the coronoid process and arch, surgeons should pay attention to the degree of segmentation (*β* = 0.349) and inward movement at the arch osteotomy site (*β* = 0.494; *r* = 0.688, adjusted *r*^2^ = 0.464, *F* = 50.412; *p* < 0.001).

**Conclusions:**

Surgical factors related to malarplasty affect the movement of specific parts of the zygoma. In addition, accurate application is possible by considering the anatomical structure of the application area when using the bending plate.

## Background

The zygomatic bone (hereinafter zygoma) determines face width and the convexity of the lower orbit to provide an oval-shaped face [1–3]. Maxillofacial surgeons have used various methods for malarplasty to reduce cheekbone prominence [4–6]. The most commonly used technique is L-shaped osteotomy [4–15], which provides superior reduction of the malar eminence compared with other techniques and facilitates fixation of the eminence and the arch. In addition, use of a pre-bending plate enables precise control of the degree of bone movement [3, 10, 13, 16].

However, surgeons subjectively determine the need for eminence segmentation during L-shaped osteotomy. Previous studies have not involved quantification of the effects of eminence segmentation [6, 7, 10, 12, 13]. In addition, the use of a standardized pre-bending plate does not improve prediction accuracy regarding the degree of arch or eminence reduction relative to surgeons’ subjective evaluations [3, 10, 13, 16]. Such plates are placed perpendicular to the tangential plane of the fixation position, leading to differences between the degree of bending and the actual three-dimensional (3D) bone movement depending on the surface curvature at the fixation point.

We quantitatively analyzed the effects of bone segmentation and plate bending on the 3D movement of the zygoma using computed tomography (CT). In addition, we evaluated the effect of the postoperative arch position at the osteotomy site on zygomatic movement.

## Methods

### Study design and sample

#### Patient selection

From zygoma reduction malarplasty procedures performed between December 6, 2019, and May 25, 2020, we selected 172 operations performed in 86 patients whose bony landmarks were clearly visible after surgery. We excluded patients who underwent re-operation. The study sample comprised 8 males and 78 females with a mean age at the time of surgery of 29.3 (range, 18–47) years. Twenty-nine patients underwent malarplasty, 11 underwent angle reduction, 44 underwent angle reduction and genioplasty, 1 underwent genioplasty, and 1 underwent double jaw surgery. The study protocol was approved by the Public Institutional Bioethics Committee (P01-202006-21-029). The study was performed in accordance with the Declaration of Helsinki. CT was performed before and within 2 days after surgery.

#### Operation method (Fig. 1)

**Fig. 1:**
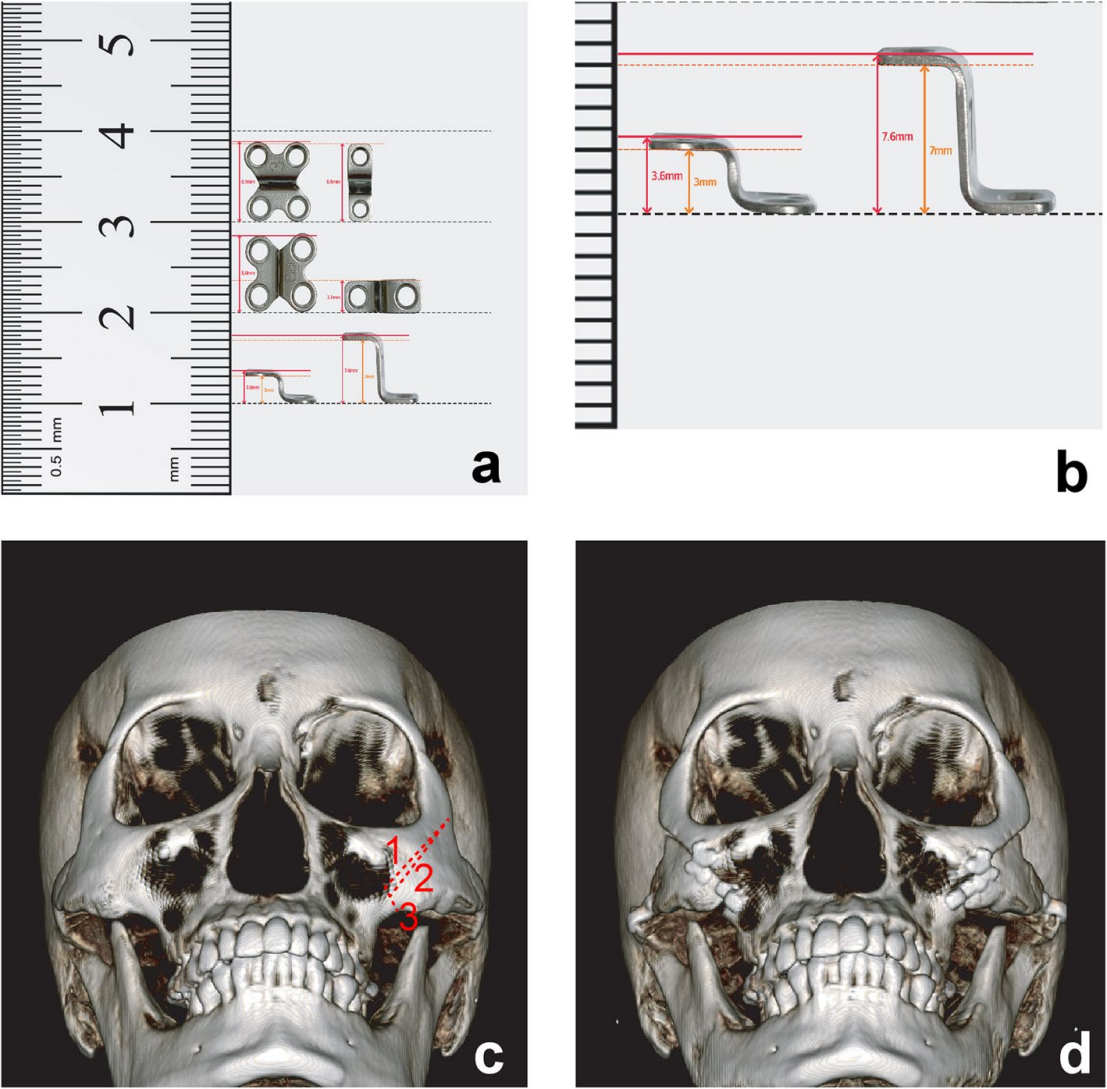
Degree of plate bending and segmentation. **a** Four-hole plate for the eminence and two-hole plate for the arch. **b** Measurement of bending degree. **c**, **d** Degree of eminence segmentation, indicated by the linear distance between osteotomies 1 and 2 at the intersection with the cut line

A conventional intraoral vestibular incision was made to create an L-shaped segmental design with narrow upper and wider lower parts [10]. The design extended from the lower lateral side of the zygomaticofacial foramen to the upper medial side of the frontal process of the zygomatic bone. The trapezoidal design enabled determination of the level of segmentation along the inferior horizontal cutting line using a micro-compass (Fig. 1c). After intraoral anterior osteotomy, the arch was exposed using a sideburn incision and an oblique incision was made in front of the articular eminence (or tubercle). The arch was cut and fixed in an advanced position according to the planned size of the bending plate (Fig. 1a and b). The operator verified a tight fit by pushing the movable bony malar eminence inward through the previously made intraoral incision. A saw was used to reduce the bony gap formed by bony interference in the upper half of the bone. In some cases, additional sawing was performed despite appropriate fitting, with the length of segmentation increased by 1 mm with each application [17]. After confirmation of a uniform fit, a four- or six-hole plate was placed between the free zygoma and the untreated medial bone (Fig. 1d). The eminence and arch were fixed using a midplate (BioMaterialsKorea, Seoul, Korea) and 1.7-mm screws (Stryker, Kalamazoo, MI, USA).

#### Anatomical landmarks

##### Malar eminence and tubercle (Fig. 2)

To improve the reproducibility of measurements in the evaluation of 3D changes in anatomical landmarks, we defined the landmarks. The malar eminence was identified as the most protruded part of the zygomatic bone when viewed from 45°. The most protruded part of an anatomical tissue is a nonmetric trait that is difficult to accurately compare before and after surgery [18]. In particular, the 45° positioning of the object is inaccurate. Several methods enabling the accurate comparison of landmarks, including palpation and CT, have been evaluated, but the identification of the target area remains subjective [19–21]. In the present study, we evaluated the anatomical structure of the malar tubercle (MT), which is easily visualized at the malar eminence site on CT scans. The MT, also known as the zygomatic hook, is an inferior projection of the maxilla and zygoma at the zygomaticomaxillary suture. The malar eminence or zygomatic prominence are identified using the zygomaticomaxillary suture [19–22]. In the present study, we measured the movement of the MT to identify the anteroposterior (AP) and vertical movements of the malar eminence. We excluded cases with relatively undeveloped MTs.

**Fig. 2:**
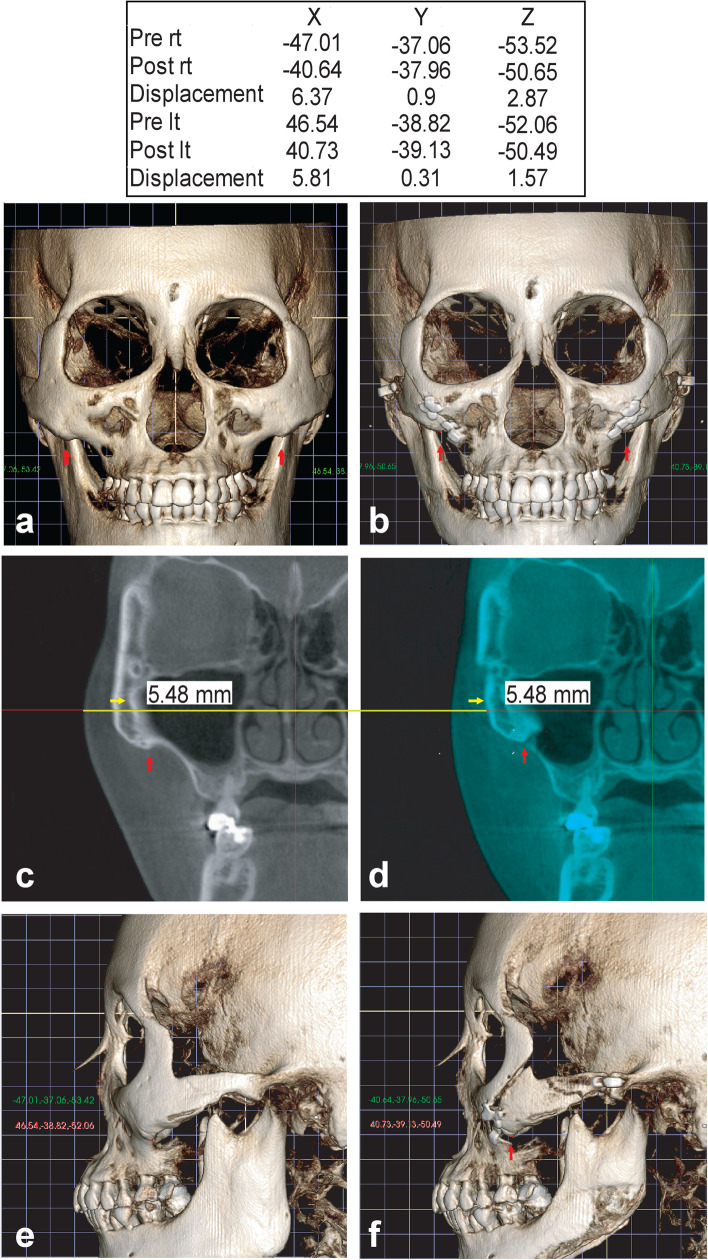
Measurement of the eminence landmark before and after surgery. Red arrows indicate the malar tubercle (MT). **a**, **b** Frontal three-dimensional (3D) images obtained before and after malarplasty. The inset table in **a** shows the 3D coordinates of the MT on each side. Pre: before surgery, post: after surgery. Positive displacement values indicate inward movement on the *x* axis, anterior movement on the *y* axis, and upward movement on the *z* axis. **c**, **d** Measurement of the inward movement of the malar eminence at the level of the most protruding soft tissues in the coronal plane, including the MT. Yellow arrow and 5.48 in **d** indicate the direction and degree of medial movement of the area. **e**, **f** Lateral 3D images of malarplasty

##### Zygomatic arch and coronoid process (Fig. 3)

Medial movement of the arch after surgery may limit its opening due to interference with the coronoid process. In the present study, we evaluated the arch movement around the coronoid process.

**Fig. 3:**
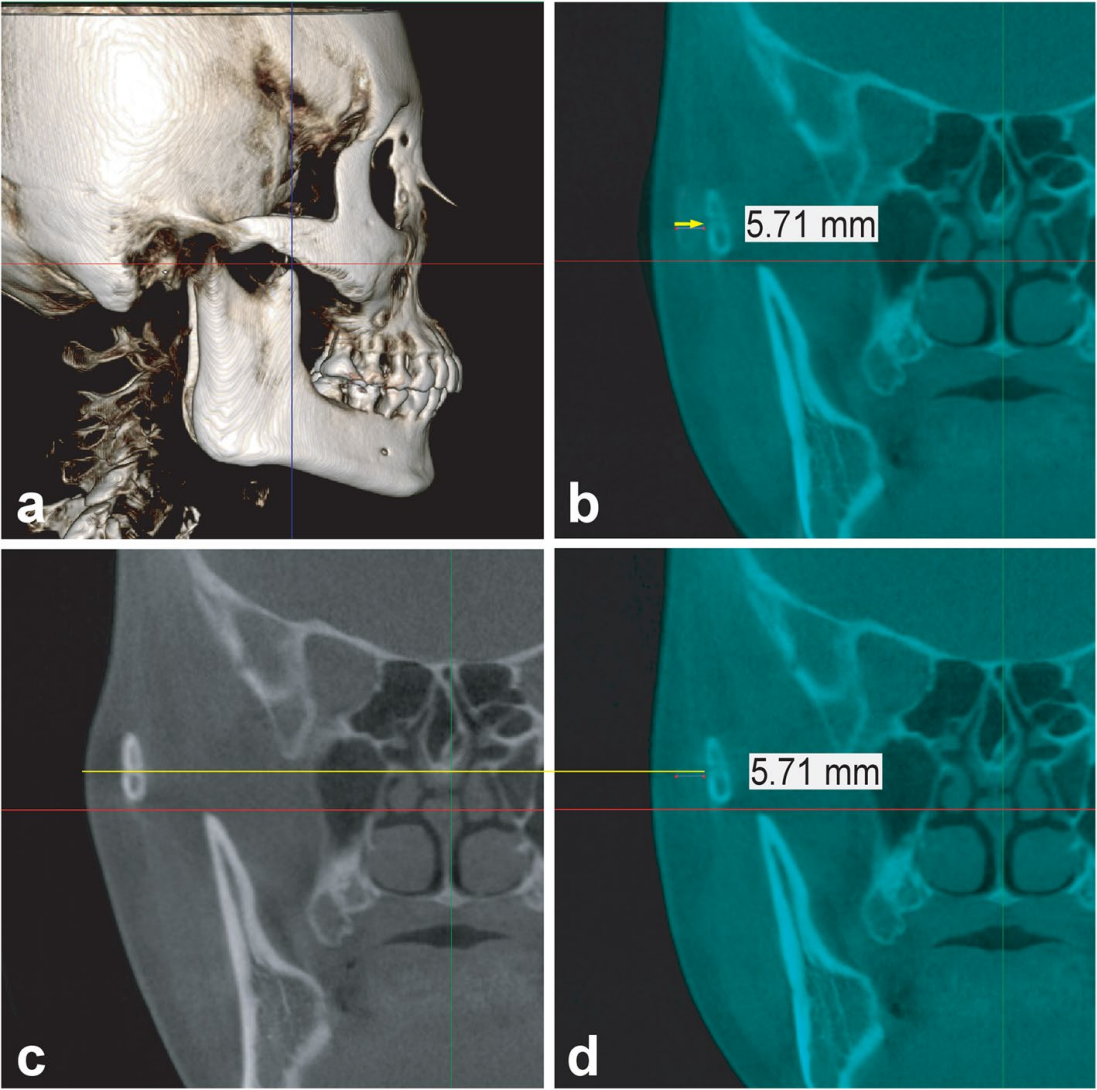
Measurement of inward arch movement at the coronoid process in the coronal plane including the apex of the process. **a** Identification of the coronoid process apex. The yellow arrow in **b** and the number below the arrow indicate the direction and degree of horizontal reduction. The yellow lines in **c** and **d** show the most abundant soft tissue. Arch reduction was confirmed at the level of the soft tissue, including the apex, in the coronal plane

#### Measurement method

CT (Alphard-3030; Asahi Roentgen, Kyoto, Japan) was performed with the use of the habitual intercuspal position. The C mode was used to obtain sufficient exposure areas. The data were processed using InVivoDental 3D imaging software (Anatomage, Inc., San Jose, CA, USA). Before analysis, the 3D coordinate system was oriented using preoperative CT images to ensure symmetry between the left and right orbital rims in the sagittal plane when the image was focused on nasion.

##### Horizontal reduction of the malar eminence (Fig. 2c and d)

The degree of horizontal bone reduction was investigated at the level of the most prominent soft tissue in the preoperative coronal plane, including the MT. The preoperative and postoperative images were superimposed for the detection of inner or medial movement.

##### Degrees of AP and vertical MT movement (Fig. 2a, b, e, and f)

After orienting the postoperative CT images using the same 3D coordinate system applied for the preoperative images, the MT had the same coordinates on the preoperative and postoperative images. AP, vertical, and forward + upward movements are indicated by the *y* value, *z* value, and “+,” respectively [15].

##### Horizontal reduction of the arch at the coronoid process (Fig. 3)

After identifying the preoperative position of the coronoid apex on CT images acquired in the maximal intercuspal position, the postoperative CT image on the preoperative image was superimposed to determine the degree of inward movement of the zygomatic arch, at the level of the most abundant soft tissue in the coronal plane including the apex of the coronoid process.

##### Change in arch position at the arch osteotomy site (Figs. 4 and 5)

**Fig. 4:**
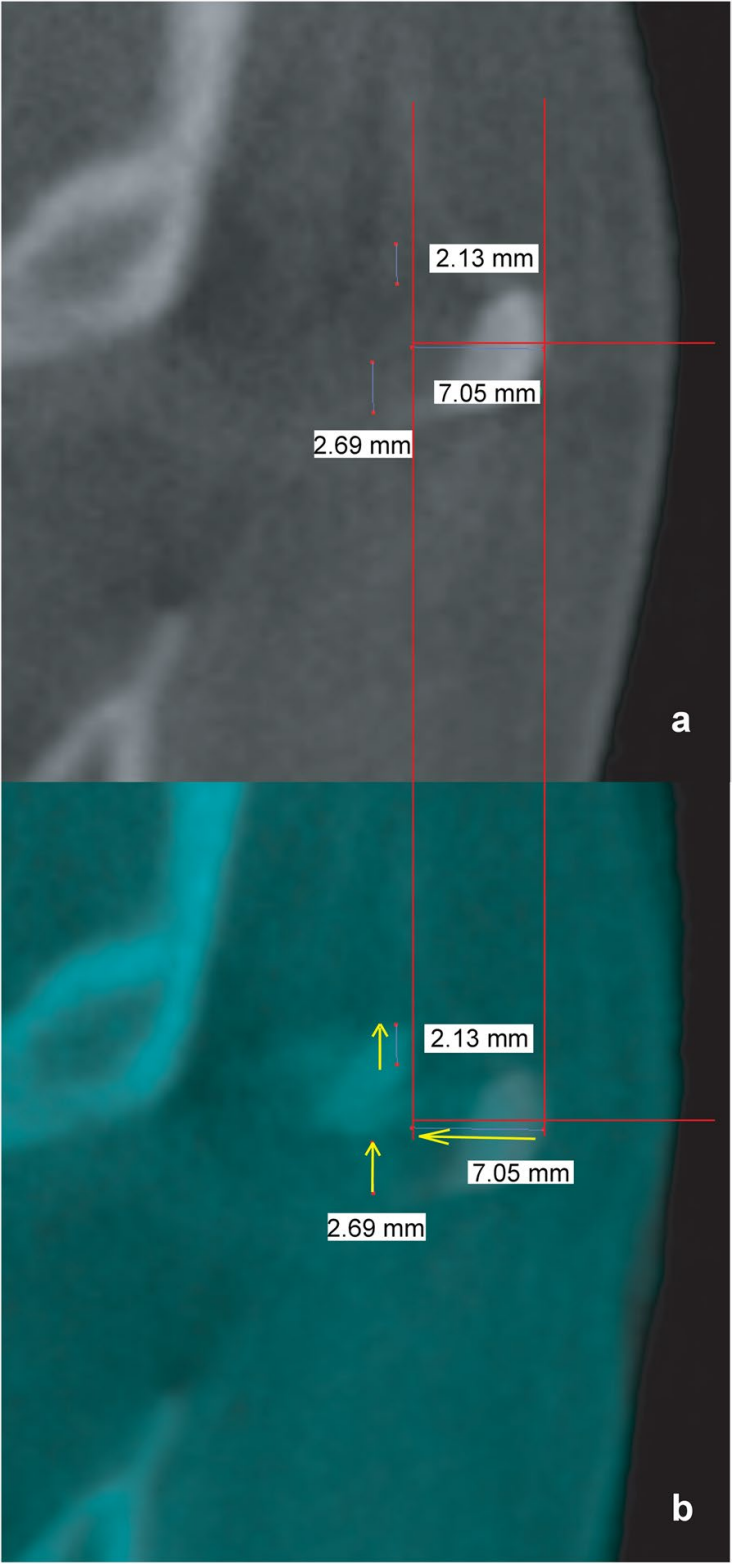
Measurements of the vertical and inward movement of the zygomatic arch on each side in the coronal plane. The investigator averaged the vertical changes at the upper and lower edges. A positive value indicates that the postoperative height of the zygomatic bone exceeded the preoperative height. Values are presented in yellow. The yellow arrow indicates the direction of postoperative zygomatic arch movement

**Fig. 5:**
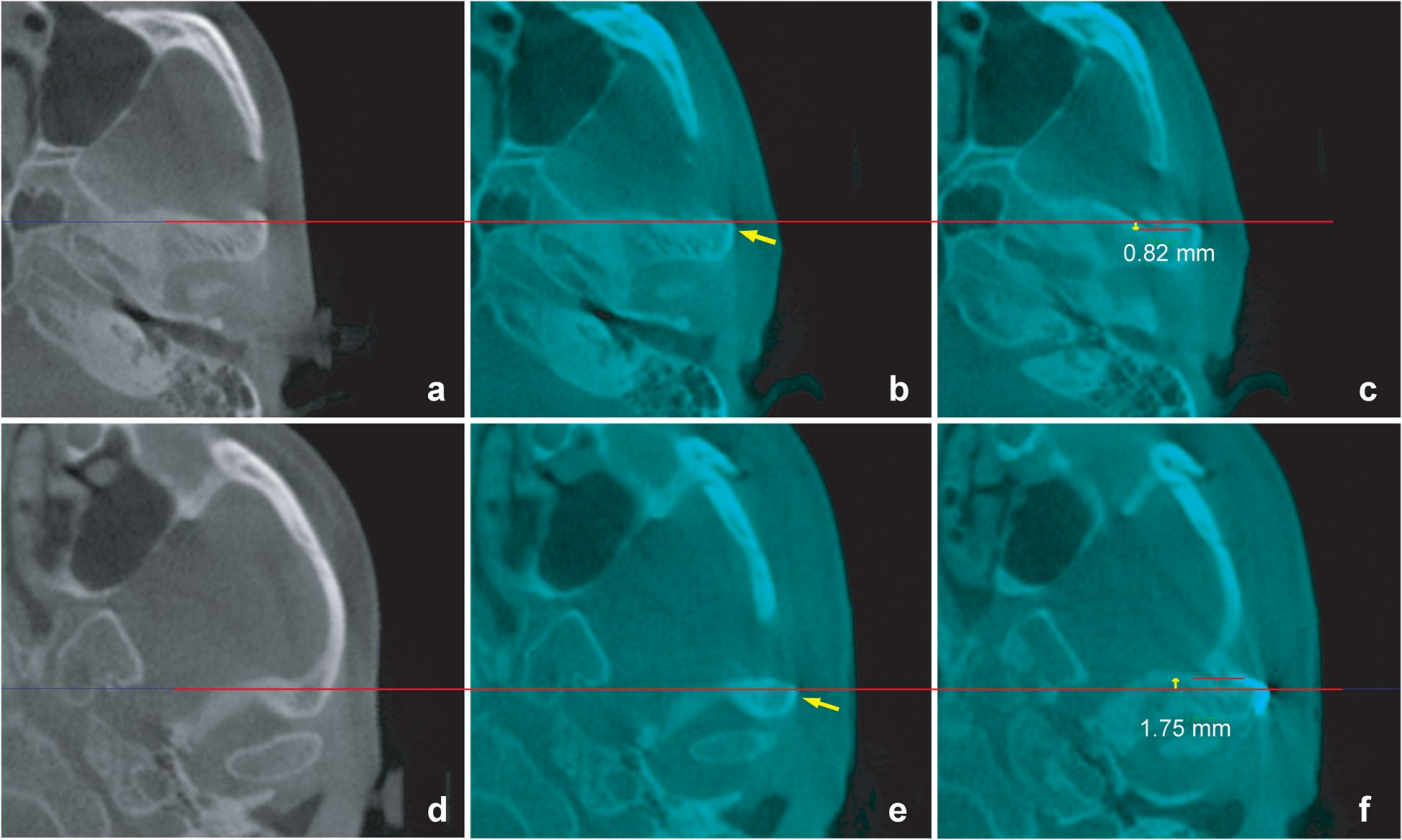
Measurement of the AP movement of the arch cutting edge on axial CT images. Comparison of the preoperative images (**a** and **d**) with the postoperative images (**b** and **e**) at the same level. The lateral cutting edge of the temporal bone is shown in the lowermost axial plane (yellow arrows in **b** and **c**). The cutting edge of the arch moved inward. When the cut edge moved posteriorly, a negative value was assigned; when it moved anteriorly, a positive value was assigned. The red line indicates the cutting edge baseline of the temporal bone. **c** and **f** show backward and forward movements of the arch, respectively

Routine arch osteotomy involves the creation of an oblique incision with the lower and upper parts of the incision extending anteriorly and posteriorly, respectively. The vertical and medial movement of the arch in the coronal plane and AP movement in the axial plane was investigated. The vertical and medial movements were determined by overlapping the preoperative and postoperative views of the arch in the coronal plane anterior to the screw hole (Fig. 4). The AP movement of the arch was measured at the level of the lower edge of the cut arch on axial images (Fig. 5).

#### Reliability of measurements

The CT images were enlarged to a predetermined size and the lengths were measured twice using an instrument provided in the InVivoDental program. The investigators viewed the CT images at × 5 magnification, such that a length of 10 cm was displayed as 2 cm on the screen. The measurements were repeated until two successive measurements had a discrepancy error of < 0.2 mm.

#### Statistical analysis

Descriptive statistical analyses of the preoperative and postoperative measurements were performed using SPSS for Windows (version 21; IBM Corp., Armonk, NY, USA). The Kolmogorov–Smirnov test was used to determine the normality of the data distribution. The degrees of plate bending and segmentation in the malar eminence served as independent variables [16]. Inward movement of the malar eminence, AP and vertical movements of the MT, inward movement of the arch around the coronoid process, and inward and vertical movements of the arch at the osteotomy site served as dependent variables.

Parametric analysis of variance (ANOVA) and the nonparametric Kruskal–Wallis *H* test were used to detect significant differences in dependent variables according to the independent variables.

### Correlation analysis

Correlations between variables were examined using parametric (Pearson) or nonparametric (Spearman) tests, as appropriate.

### Multiple regression analysis

In multiple regression analyses, the degree of segmentation; degree of plate bending; and upward, inward, and AP arch movements at the arch osteotomy site served as independent variables. Multiple regression analyses were performed to evaluate the effects of the independent variables on the inward movement of the malar eminence, AP movement of the MT, and inward arch movement at the coronoid process. A stepwise elimination method was used to estimate regression coefficients. The Durbin–Watson test was used to confirm the absence of autocorrelation and multicollinearity (variance inflation factor ≥ 10). No outliers were detected.

## Results

Table 1 presents the malar eminence movement according to the degree of plate bending and segmentation at the eminence. The mean amount of inward movement was 4.57 ± 1.06 mm. Plates bent 0 and 4 mm showed 3.46 ± 1.27 and 4.54 ± 1.15 mm inward movement, respectively. The mean amount of backward movement was − 0.62 ± 1.22 mm. Plates bent 0 and 4 mm showed 0.58 ± 0.76 and − 1.25 ± 1.30 mm AP movement, respectively. The mean amount of vertical MT movement was 2.49 ± 1.04 mm. Plates bent 0 and 4 mm showed 2.46 ± 0.78 and 2.67 ± 1.11 mm vertical movement, respectively. The inward movement of the malar eminence and AP movement of the MT differed significantly according to the degree of plate bending (*p* = 0.000, ANOVA for inward movement; *p* = 0.000, Kruskal–Wallis *H* test for AP movement). The 2-mm and 6-mm segmentations were associated with 2.30 ± 0.50 and 5.71 ± 0.88 mm inward movement of the eminence, respectively, and 0.58 ± 1.11 mm anterior and − 1.03 ± 1.37 mm posterior MT movement. After 2-mm and 6-mm segmental osteotomies, the amounts of vertical MT movement were 1.92 ± 0.74 and 2.76 ± 1.17 mm, respectively. This analysis confirmed significant differences in the degrees of inward and AP MT movement according to the degree of segmentation (*p* = 0.000, ANOVA for inward movement; *p* = 0.035, Kruskal–Wallis *H* test for AP movement).

**Table 1:** Movement of the malar eminence according to the degree of plate bending and segmentation at the eminence

			Inward movement at the zygomatic eminence		AP movement of MT		Vertical movement of MT	
		*N*	Mean	SD	Mean	SD	Mean	SD
Degree of plate bending at the zygomatic eminence	0	12	3.46	1.27	0.58	0.76	2.46	0.78
	2	48	4.18	0.84	− 0.12	1.15	2.25	1.2
	3	94	4.83	0.97	− 0.9	1.11	2.57	0.94
	4	18	4.96	1.15	− 1.25	1.3	2.67	1.11
Degree of body segmentation	2	6	2.3	0.5	0.58	1.11	1.92	0.74
	3	26	3.98	0.76	− 0.54	1.37	2.31	1.1
	4	56	4.14	0.84	− 0.43	1.13	2.34	0.86
	5	67	5.06	0.75	− 0.8	1.14	2.66	1.1
	6	17	5.71	0.88	− 1.03	1.37	2.76	1.17
Average		172	4.57	1.06	− 0.62	1.22	2.49	1.04

*MT* malar tubercle

*+*, anterior or upward movement

Inward movement at the zygomatic eminence: measurements of bone changes from baseline after surgery in the coronal plane in the region with the most protruding soft tissues, including the MT, before surgery

AP movement of the MT: the degree of AP movement of the MT

Vertical movement of the MT: the degrees of upward and downward movement of the MT

Table 2 presents the zygomatic arch movement according to the degrees of plate bending and segmentation at the eminence. For 0 and 4 mm plate bending at the eminence, the amount of inward arch movement at the coronoid process was 4.99 ± 1.24 and 5.22 ± 0.94 mm, respectively. For 0- and 4-mm plate bending, the amount of vertical movement at the arch osteotomy site was 3.36 ± 1.33 and 4.31 ± 1.59 mm, respectively. ANOVA confirmed that plate bending was related significantly to vertical movement at the arch osteotomy site (*p* = 0.012), but not to inward movement. For 0- and 2-mm segmentation, the amount of inward arch movement at the level of the coronoid process was 3.79 ± 0.48 and 5.59 ± 1.0 mm, respectively; the amount of inward arch movement at the arch osteotomy site was 5.69 ± 1.08 and 6.68 ± 0.80 mm, respectively. ANOVA revealed significant differences in inward arch movement at the coronoid process and the arch osteotomy site according to the degree of segmentation (*p* = 0.000 and *p* = 0.001, respectively). No such difference in the vertical movement of the arch at the arch osteotomy site was observed. With 5- and 9-mm plate bending, the amount of inward arch movement at the arch osteotomy site was 4.48 ± 0.29 and 7.21 ± 0.82 mm, respectively. The use of plates bent 5 and 9 mm resulted in postoperative changes in the arch height of 3.66 ± 0.36 and 3.9 ± 1.44 mm, respectively, with a significant difference between them (ANOVA: *p* = 0.000 for inward movement, *p* = 0.047 for vertical movement).

**Table 2:** Movement of the malar arch according to the degree of plate bending and eminence segmentation

			Inward movement at the coronoid process		Inward movement at the arch osteotomy site		Vertical movement at the arch osteotomy site		AP movement at the arch osteotomy site	
		*N*	Mean	SD	Mean	SD	Mean	SD	Mean	SD
Degree of plate bending at the zygomatic eminence	0	12	4.99	1.24	6.65	0.99	3.36	1.33	0.34	1.01
	2	48	5.08	0.83	6.54	1.01	3.21	1.22	0.33	0.64
	3	94	4.9	1.01	6.25	0.98	3.65	1.15	0.47	0.86
	4	18	5.22	0.94	6.34	0.97	4.31	1.59	0.53	0.85
Degree of body segmentation	2	6	3.79	0.48	5.69	1.08	3.24	1.08	0.77	1.01
	3	26	4.35	0.93	5.88	0.75	3.59	1.28	0.31	0.49
	4	56	4.74	0.84	6.24	0.95	3.5	1.3	0.63	0.95
	5	67	5.4	0.8	6.65	1.04	3.57	1.14	0.28	0.66
	6	17	5.59	1	6.68	0.8	3.96	1.66	0.44	1.06
Degree of plate bending at the zygomatic arch	5	3	3.83	0.95	4.48	0.29	3.66	0.36	0.33	0.58
	6	9	4.11	0.8	5.17	0.59	3.96	1.18	0.38	0.52
	7	46	4.6	0.75	5.59	0.54	3.18	1	0.33	0.58
	8	56	5.05	0.89	6.43	0.61	3.49	1.22	0.3	0.57
	9	58	5.44	0.98	7.21	0.82	3.9	1.44	0.64	1.13
Mean		172	4.99	0.97	6.37	0.99	3.57	1.26	0.43	0.81

+, anterior or upward movement

Correlation analysis revealed correlations between surgical variables and postoperative bone landmarks. The medial movement of the malar eminence correlated moderately with the degree of body segmentation (Pearson’s *r* = 0.650, *r*^2^ = 0.422). The medial movement of the arch at the coronoid process correlated with the degree of segmentation and arch plate bending (*r* = 0.490 and *r* = 0.397, respectively). Inward movement at the arch osteotomy site correlated strongly with the degree of plate bending at the zygomatic arch (*r* = 0.738, *r*^2^ = 0.545).

### Multiple regression analysis results

Factors affecting inward movement of the malar eminence: Table 3 presents the degrees of eminence segmentation and plate bending at the malar eminence [adjusted *r*^2^ (coefficient of determination) = 0.479]. The Durbin–Watson value (1.466) confirmed the absence of autocorrelation. The explanatory power (*r*^2^) of the regression equation was meaningful (*p* = 0.000).

**Table 3:** Multiple regression analysis results

	Affecting factor	*B*	SE	*β*	*t*	*p*	
Inward movement of the malar eminence	Degree of eminence segmentation	0.645	0.061	0.593	10.48	0	*r* = 0.696, adjusted *r*^2^ = 0.479 Durbin-Watson value = 1.466 *F* = 79.595 *p* < 0.001
	Degree of plate bending at the zygomatic Eminence	0.292	0.064	0.257	4.545	0	
AP movement of the malar tubercle	Degree of plate bending at the zygomatic eminence	− 0.464	0.082	− 0.354	− 5.633	0	*r* = 0.603 adjusted *r*^2^ = 0.352 Durbin-Watson value = 1.781 *F* = 31.943 *p* < 0.001
	Degree of vertical movement at the arch osteotomy site	− 0.368	0.062	− 0.379	− 5.947	0	
	Degree of AP movement at the arch osteotomy site	0.489	0.095	0.324	5.171	0	
Inward movement of the arch at the coronoid process	Degree of body segmentation	0.348	0.059	0.349	5.935	0	*r* = 0.688 adjusted *r*^2^ = 0.464 Durbin–Watson value = 1.555 *F* = 50.412 *p* < 0.001
	Degree of inward movement at the arch osteotomy site	0.484	0.058	0.494	8.411	0	
	Degree of vertical movement at the arch osteotomy site	− 0.092	0.043	− 0.119	− 2.124	0.035	

Factors affecting AP movement of the MT: We found that the upward and posterior movements of the arch had main effects on the anterior and posterior movements of the eminence, respectively (adjusted *r*^2^ = 0.352, Durbin–Watson value = 1.781).

Factors affecting inward arch movement at the coronoid process: Table 3 shows the mesial arch movement, degree of segmentation, and vertical arch movement (adjusted *r*^2^ = 0.464, Durbin–Watson value = 1.555).

## Discussion

Although the effects of several techniques for malarplasty have been examined, the main outcomes assessed have been self-reported patient satisfaction [6, 10, 13, 16] and operator evaluations of the changes in eminence projection and oval shape of the face [7].

Few studies have involved the quantitative evaluation of zygomatic movement using CT images obtained after malar reduction surgery [3, 15, 17, 23]. Existing reports describe the surgical outcome in a single patient based on CT findings [3] and the malar eminence reduction after bony segment removal from the anterior portion of the zygoma without arch cutting or internal fixation [23]. In two other studies in which osteotomy and rigid fixation were performed for malar eminence and arch alteration, no detailed description of the surgical method for the arch was provided and only the surgical procedure and outcome for the eminence were evaluated [15, 17]. Such an approach does not account for the possible relation between the malar eminence and arch. The surgical factors related to the two surgical sites may have combined effects on the final position of the zygoma after malarplasty. This is the first study in which the effects of intraoperative inward, AP, and upward arch movements on surgical outcomes were evaluated.

We used different bony landmarks than previous studies. The summit of the zygoma, including the zygomaticotemporal suture, has been used as an arbitrary plane for the measurement of the inner movement of the malar eminence [17]. However, the position of the zygomaticotemporal suture, used as the reference point for this plane, may change after surgery [17]. In another study, the lowest point of the zygomaticotemporal suture was used to measure the AP movement of the malar eminence [15]; this landmark is useful for the identification of changes in the malar eminence. In the present study, we used the MT as a bony landmark because it projects more visibly than the zygomaticomaxillary suture and is easily measured. In contrast, the zygomatic bone is round, which makes identification of a specific point on the suture difficult. Additionally, the zygomatic bone is more easily differentiated in Asians than in Europeans [22]. However, our personal experience suggests that, although the zygoma is clinically prominent at 45°, some patients have underdeveloped MTs, particularly when the maxillary sinus is bulging. Such cases were excluded from the present study.

The use of bony landmarks to measure changes in the malar eminence is controversial. Conflicting results of the use of anatomical landmarks for CT image analysis have been reported [3, 15, 17, 23]. Additionally, even though CT data on the same patient may be reconstructed by orienting the view at 45° using 3D analysis software, the use of a reference for orientation is controversial [19–21].

In this study, we compared preoperative and postoperative protrusion on CT using the same cross-sectional image-based coordinates, in contrast to other studies [15, 17]. Facial narrowing may be detected on the basis of horizontal (*x*-axis) changes in bony landmarks, such as zygion, defined as the most protruding part in the frontal view. Because the position of zygion may change postoperatively, changes in the three-coordinate *x* value for zygion do not indicate facial narrowing. In a previous study, the preoperative and postoperative positions of the malar eminence in the same cross-sectional plane were evaluated, and the arch width and eminence positions were compared by overlaying preoperative and postoperative images of the same cross-section [23].

We investigated the correlation between three surgical factors (degrees of bone segmentation and plate bending at the malar eminence and the arch) and the postoperative locations of bony landmarks. The only significant correlation was between the degree of plate bending and inward arch movement at the arch osteotomy site (*r*^2^ = 0.545). Additionally, we performed multiple regression analysis of the surgical factors identified on CT images.

The multiple regression analysis of inward movement at the malar eminence showed that the degree of bone segmentation at the eminence was the main factor involved in the inward movement of the eminence, consistent with previous findings [17, 23]. Some surgeons avoid segmentation to improve postoperative functional stability; however, our results indicate that the horizontal reduction of the malar eminence is of limited usefulness without segmentation. Thus, our results differed from previous reports of excellent reduction of the eminence, even when the malar bone was cut without body strip removal [12, 13, 15, 24].

Our results differ from those of previous quantitative analyses of the zygoma position on postoperative CT images. We found that the degree of bone segmentation was related to the medial and posterior movements of the malar eminence. Conversely, a previous study showed that the direction of movement was forward [17]. This discrepancy may be related to differences in the measurement method and surgical procedure. In the previous study, the summit of zygoma was used to measure the positional changes, whereas we measured the postoperative changes in the MT position. In the previous study, the surgeon fixed the body or eminence before the arch was fixed, whereas the arch was fixed first in the present study. Additional research is needed to determine the optimal fixation sequence.

**Fig. 6:**
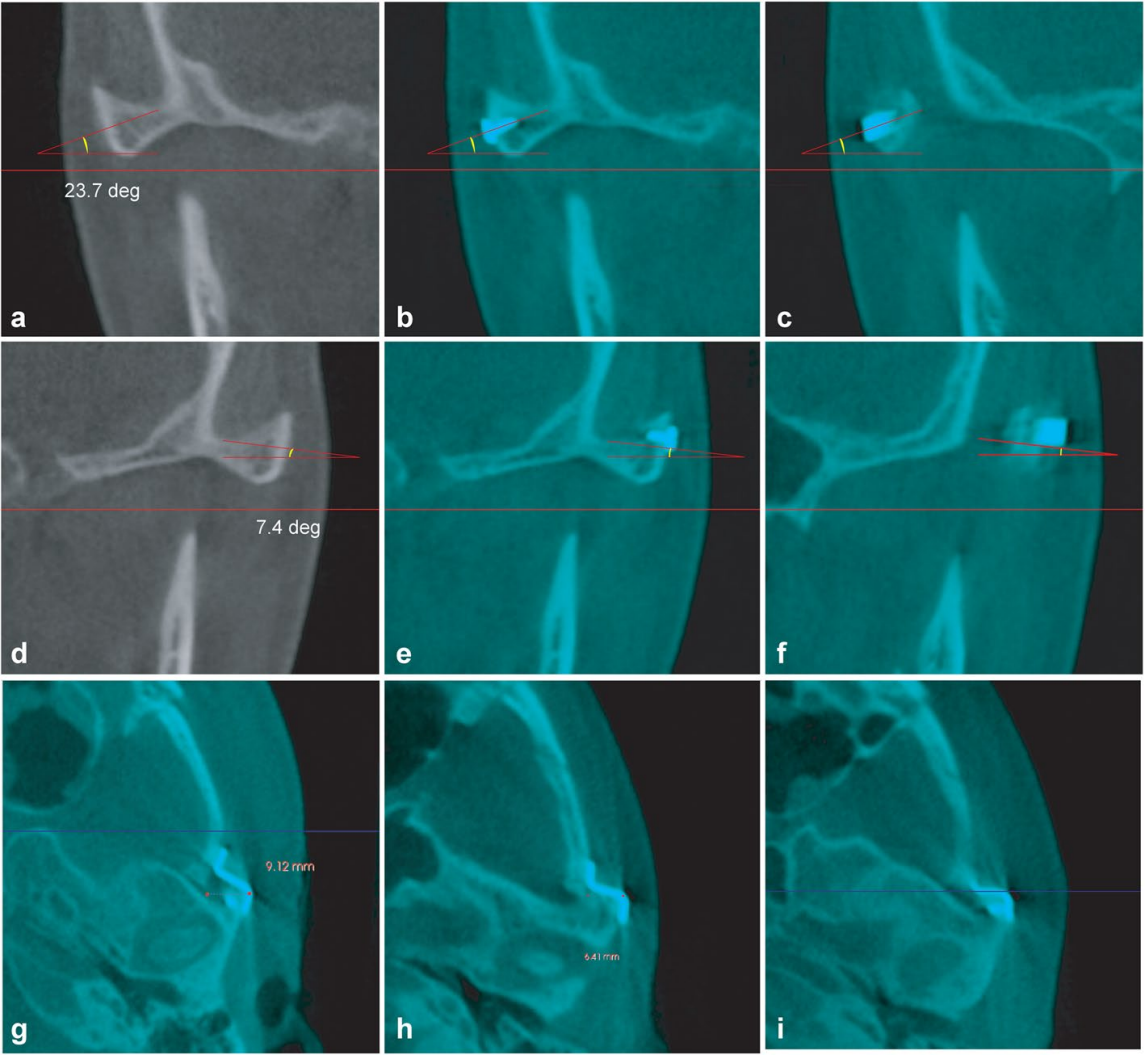
Insufficient inward movement of the arch with degree of arch plate bending. **a**–**f** Inclination of the arch in the coronal plane leading to upward arch fixation. **g** Anterior arch movement due to interference of the arch with the temporal bone. **h**, **i** Anterior arch movement according to the curvature of the cut site

We found that the postoperative arch location has a greater effect on AP movement than does the degree of plate bending at the eminence. In contrast to previous studies, in which changes in arch-related surgical factors were not considered [15, 17], we evaluated the effect of upward arch fixation on the backward movement of the zygoma (Fig. 6).

The multiple regression analysis showed that the application of the eminence bending plate affected the inward and AP movements of the malar eminence. The inward movement was related to the tetrapodal shape of the zygomatic bone, which has a narrow outer side and a broad inner side [25]. The step between the mobilized eminence and the untreated zygoma, including the orbit, results from the internal rotation of the eminence around a concentric center determined by arch fixation. In other words, the operator passively fitted the bending plate to the bony step, and the plate may not have induced the backward movement [23]. The operator selected the bending plate size corresponding to the step. This inference is supported by the fixation order, namely the fixation of the arch before the eminence.

Interference between the arch and the coronoid process can result in limited mouth opening after malarplasty. Thus, the surgeon should be cautious regarding the degrees of segmentation and plate bending at the arch, particularly when the coronoid process and arch are close to each other on preoperative CT images.

Inward arch movement correlated strongly with the arch plate bending size (*r* = 0.738, *r*^2^ = 0.545). The midface width could be reduced by cutting the arch and pre-bending the plate without resectioning the zygomatic body, in line with previous findings [16].

However, the inward movement of the arch did not accurately reflect the corresponding plate bending size, which may be explained by the surface anatomy of the zygoma. Surgeons probably expect the bending plate used for the arch to be involved only in inward arch movement. However, the diversity of arch shapes can change the 3D placement of the plate (Fig. 5). Thus, the 3D shape of the arch osteotomy site must be considered before surgery. The curvature or inclination of the fixation site affects the differences in bone movement on the *x*, *y*, and, *z* axes [17, 25]. The upper part of the arch is located laterally and the lower part is located mesially. This shape accounts for the bony inclination in the coronal plane. Greater bony inclination is associated with greater upward arch movement and reduced inward movement after fixation (Fig. 6a–f). Greater vertical inclination of the arch is associated with greater medial movement.

The arch is widest in front of the articular eminence, and its curvature in the axial plane accounts for the anterior arch movement induced by the bending plate after fixation. Figures 6g–i show the anterior locations of the arch according to the curvature of the arch. The diversity in arch shape enables the use of a bending plate, which affects inner, upward, and forward arch movements. As a result, the degree of bending does not accurately reflect the inward movement of the arch.

The most accurate technique for malarplasty involves the use of a customized surgical stent [26, 27]. However, this technique is time consuming and expensive. Additionally, the bone in the anterior maxillary wall is thin, rendering fixation of the metal plate in the desired position difficult. Furthermore, extensive dissection is required for stent use, which can cause the cheeks to droop. Thus, the surgical variables that affect outcomes after application of the currently available surgical techniques must be identified.

The ultimate goal of reduction malarplasty is esthetic soft tissue changes. The universal use of cone beam CT has made it possible to observe soft tissue changes according to bone movement [28, 29]. However, these studies only provide information on the amount of change in soft tissue caused by the final position of the bone. Regarding the effect of internal bone movement on the horizontal reduction of soft tissue, our study is meaningful in that it analyzed the factors affecting inward bone movement in a specific area in detail [28].

Additional studies involving finite element analysis of surgical outcomes are required. We found that the bone movement due to the bending plate differed according to the curvature of the zygoma, which should be considered in future such studies.

## Conclusions

We quantitatively analyzed the effects of bone segmentation and plate bending on the 3D movement of the zygoma using CT. Additionally, we evaluated the effect of the postoperative arch position at the osteotomy site on zygomatic movement. Segmentation had the most significant effect on the horizontal reduction of malar eminence. Additionally, upward and posterior arch movements had significant effects on the anterior and posterior movements of the eminence. Notably, bone movement due to the plate bending did not accurately reflect the corresponding degree of plate bending. By accounting for the anatomical structure of the application area when using a bending plate, accurate application is possible, which should be considered in zygoma reduction malarplasty.

## Data Availability

The datasets used and/or analyzed during the current study are available from the corresponding author on reasonable request

## Abbreviations

CT: Computed tomography
MT: Malar tubercle
AP: Anteroposterior
ANOVA: Parametric analysis of variance
